# Pattern-pulses and pattern-reversals evoke different cascades of cortical sources in the multifocal visual evoked potential

**DOI:** 10.1167/jov.25.7.1

**Published:** 2025-06-02

**Authors:** Kieran S. Mohr, Anna C. Geuzebroek, Simon P. Kelly

**Affiliations:** 1 School of Electrical and Electronic Engineering and UCD Centre for Biomedical Engineering, UCD Engineering and Materials Science Centre, University College Dublin, Belfield, Ireland

**Keywords:** pattern reversal, multifocal, visual evoked potential (VEP), forward-model, retinotopic map

## Abstract

The multifocal visual evoked potential (mVEP), elicited by either pattern-pulses or pattern-reversals, provides an effective means to study visual processing and to identify retinal damage and visual field defects. It is often assumed that the first components of these VEPs, the C1 and N75, respectively, are generated in V1, based on source modeling and their polarity reversal between upper- and lower-field stimulus presentations. However, limitations in the spatial resolution of source modeling and the non-uniqueness of the polarity reversal heuristic leave this assumed V1 source uncertain. We recently demonstrated the utility of a novel method to resolve visual sources by correlating retinotopically varying VEP topographies with predictions from the Benson-2014 retinotopy atlas. Here, we apply this method to study the sources of both the pulse and reversal mVEP, presented at the same stimulus event rates of between 3–8 Hz per location (approximately 35 Hz overall event rate). This analysis suggested that although V1 dominated the generation of the pulse mVEP throughout its time course, the initial component of the reversal mVEP was instead dominated by extrastriate areas, with V1 dominance emerging later from approximately 110 ms onwards. Although the initial component of the reversal mVEP did exhibit the classic sign of a V1 source—polarity reversal across the horizontal meridian—this basic feature is also produced by extrastriate areas such as V2 and V3, and the strong lateralization of topographies near the vertical meridian predicted by a V1 source was not observed. These results suggest that the pulse and reversal mVEP evoke different cascades of generative visual areas when evoked at the event rate tested here.

## Introduction

The multifocal visual evoked potential (mVEP) is often used as a clinical biomarker of visual field defects and retinal damage because it provides a comprehensive retinotopic map of VEP responses allowing for a detailed localization of any irregularities (Fortune, Zhang, Hood, Demirel, & Johnson, 2004; Graham, Klistorner, & Goldberg, 2005; Hood et al., 2000; Hood, Odel, & Winn, 2003). It does this by rapidly presenting black-and-white checkerboard patterns at multiple retinotopic locations in tandem using independent stimulation sequences. Both pattern-pulse variants, where patterns appear briefly against a uniform gray background, and pattern-reversal variants, where black checks switch to white and white to black in continuously-on stimuli, of this mVEP have been used for this purpose. Comparisons between the two have largely focused on which has the better signal-to-noise ratio (SNR), as well as identifying moderating factors for SNR such as stimulus eccentricity, pulse rate, contrast response functions (CRFs) and spatial and temporal sparsity (Fortune, Demirel, & Bui, 2009; Hoffmann, Straube, & Bach, 2003; James, 2003; James, Ruseckaite, & Maddess, 2005; Klistorner & Graham, 2005; Maddess, James, & Bowman, 2005; Souza et al., 2012). Some of these moderating factors were found to affect the two mVEPs to different extents. For example, SNR improvements for central compared to peripheral stimuli are stronger for the pulse mVEP (Hoffmann et al., 2003; Klistorner & Graham, 2005; Souza et al., 2012); the CRF is flatter for the reversal mVEP (Maddess et al., 2005); SNR improvements with spatial sparsity are stronger for the pulse mVEP (Fortune et al., 2009); and whereas optimal SNR in the pulse mVEP is achieved at intermediate pulse rates (James et al., 2005; Maddess et al., 2005), to our knowledge the SNR of the reversal mVEP has only been reported at high reversal rates. Although these findings suggest differences in response profiles, it remains an open question whether there are substantive differences in generative mechanisms underlying the two mVEPs.

One way that differences in the response properties of the pulse and reversal mVEPs could emerge is via differences in their cortical sources. Studies examining the cortical sources and waveform morphologies of the pulse and reversal mVEP have tended to note similarities between them. The two waveforms have been judged to be almost identical in morphology and to differ substantively only in magnitude, which is several times larger in the pulse mVEP, mostly as a result of contrast adaptation in the reversal mVEP (Fortune et al., 2009; James, 2003). Many studies have inferred that both are generated primarily in area V1 due partly to the reversal of their polarity between the upper and lower visual fields and partly based on source modeling (Fortune et al., 2009; Hood et al., 2006; James, 2003; Slotnick, Klein, Carney, Sutter, & Dastmalchi, 1999; Souza et al., 2012; Zhang & Hood, 2004). A similar parallel has also been drawn for transiently presented stimuli (having event rates typically less than 2 Hz) where V1 has been identified as a source for both the C1 component of the pattern onset VEP (Clark, Fan, & Hillyard, 1994; Di Russo, Martínez, Sereno, Pitzalis, & Hillyard, 2002; Jeffreys & Axford, 1972; Mohr, Geuzebroek, & Kelly, 2024; but see Ales, Yates, & Norcia, 2010) and the N75 component of the pattern-reversal VEP (Barnikol et al., 2006; Di Russo et al., 2005). If the pulse and reversal mVEPs indeed have similar cortical sources, then functional differences between them may reflect differing neural mechanisms within the same cortical areas, such as the relative balance of activity in the magnocellular and parvocellular pathways (Klistorner & Graham, 2005); as such, a systematic investigation of cortical sources represents a crucial step to understand these differences.

However, there remains some level of uncertainty about the cortical sources of the reversal mVEP. For one, the polarity reversal heuristic classically used for V1 source identification has been demonstrated to also be predicted by areas V2 and V3 and so is not by itself particularly diagnostic of a V1 source (Ales et al., 2010; Mohr et al., 2024). Second, caution is also needed in interpreting source modeling results in the visual cortex, given the densely packed nature of visual cortical areas (Felleman & Essen, 1991), and issues of cross-talk where two cortical areas with similar geometric orientation can make indistinguishable predictions of signal topography (Dale & Sereno, 1993; Hauk, Stenroos, & Treder, 2022; Zorzos, Kakkos, Ventouras, & Matsopoulos, 2021). Beyond these technical limitations, hints of potential differences in cortical sources arise in comparing pulse and reversal mVEP waveforms in different studies: on one hand, the pulse mVEP waveform appears to include moments in time where all electrodes pass through zero together, suggestive of a multi-phasic response in a single visual area (James, 2003; Lalor, Kelly, & Foxe, 2012; Mohr et al., 2024) similar to what is typically seen in evoked local field potentials within a given early visual area (Kajikawa & Schroeder, 2015; Schroeder, Mehta, & Givre, 1998). Meanwhile, the reversal mVEP does not typically show this feature (Ales, Carney, & Klein, 2010; Capilla et al., 2016; Slotnick et al., 1999). Together, these considerations highlight the lingering uncertainty about the cortical sources of the pulse and reversal mVEP.

Recent studies applying advanced source modeling methods in the visual cortex have modeled VEP topographies in multiple retinotopic locations simultaneously in order to exploit the different patterns of cortical surface folding that exist in different visual areas, which offer strong model constraints that can distinguish even closely neighboring visual areas (Ales et al., 2010; Hagler, 2014; Hagler et al., 2009; Hagler & Dale, 2013). We recently applied such a method to investigate the cortical sources of multiple pattern-onset VEPs, including the pulse mVEP, reaffirming previous contentions that the pulse mVEP is mostly generated in V1 (Mohr et al., 2024). However, to date, these methods have not been applied to investigate the cortical sources of the reversal mVEP and to our knowledge, the sources of the pulse and reversal mVEP have not yet been addressed within the same study. Here, we applied this retinotopically constrained source analysis method to carry out the first joint assessment of the sources of the pulse and reversal mVEP in the same study, as well as how these sources change over the time course of each mVEP response. In so doing, we replicate our earlier finding that the pulse mVEP is dominated by a source in V1 (Mohr et al., 2024) throughout its time course, but find that the reversal mVEP was better explained in its initial phase by extrastriate sources, with V1 becoming dominant at later latencies and exhibiting a similar pattern to the pulse mVEP at a delay of approximately 50 ms. By demonstrating these divergent patterns, we identify that the cortical generators of the initial part of the pulse mVEP and the reversal mVEP are not equivalent and that these two signals may reflect different visual mechanisms. These findings provide a guide for interpreting alterations in these two forms of the mVEP with reference to their different generating cortical areas.

## Method

### Participants

Thirteen healthy young adults took part in this experiment (5 Females, all right-handed, aged between 21 and 45). They were compensated for their participation with a lump sum of €20. All participants gave written informed consent to be included in the study, were over the age of 18, had normal or corrected to normal vision and reported no neurological or psychiatric conditions. All operations were approved by the Human Research Ethics for Sciences board of University College Dublin and adhered to the guidelines set out in the Declaration of Helsinki.

### Stimuli

**Figure 1.**
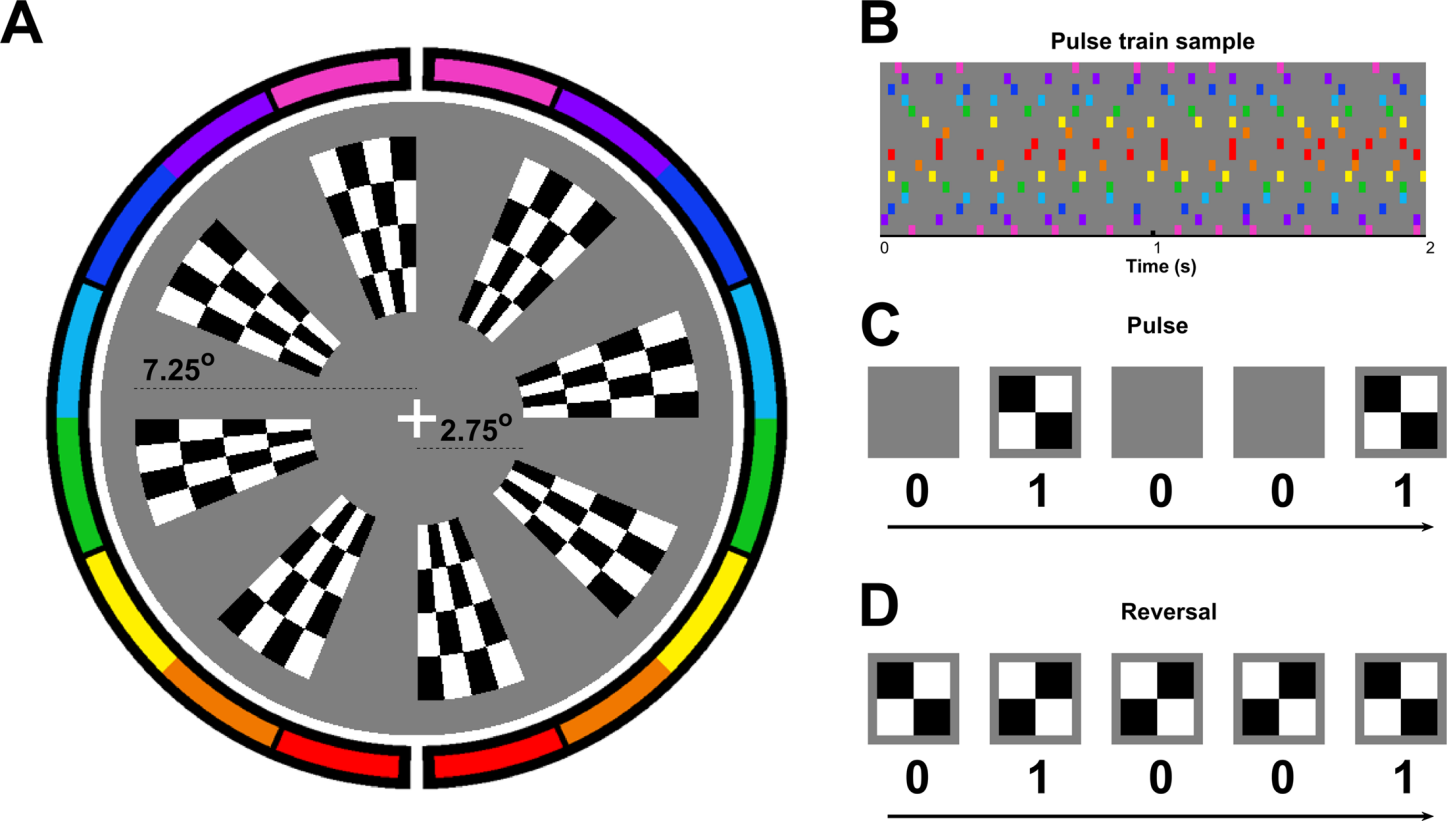
(**A**) Eight of the 16 checkerboard wedges used in both mVEP protocols with the eccentricities indicated. (**B**) Example of a pulse train (binary sequence of 1s, colors, and 0s, gray) used in both mVEP protocols, showing each location as a row, color-coded in the same way as in panel A. (**C**) In the pulse protocol, a checkerboard wedge was presented at each event (1 in the binary sequence), whereas the gray background remained on screen otherwise. (**D**) In the reversal protocol, all 16 checkerboards were always present and reversed the black-and-white pattern at each event (1 in the binary sequence).

Stimuli were identical to those used in Mohr et al (2024; see Figure 1) except that they were presented on a different monitor (*Asus TUF Gaming VG259QM G-SYNC Compatible*), which operated at a refresh rate of 240 Hz. Participants were seated at a chin rest in a dark sound-attenuated chamber with no other task than to maintain a stable gaze on a white fixation cross at a distance of 57 cm while stimuli were presented to them using Psychtoolbox-3 (Brainard, 1997; Kleiner et al., 2007; Pelli, 1997). When stimuli were not presented, the background was maintained as a uniform gray field at 45 cd/m^2^ (half the monitor's maximum luminance). The stimuli included 16 equal-sized wedge segments that partitioned an annular “dartboard” pattern of alternating black and white checks (4 × 4), with a radius range of 2.75° to 7.25° (see Figure 1A). The retinotopic location of each wedge was determined by its polar angle, with the first located between 0° and 22.5°, the second between 22.5° and 45°, and so forth. The retinotopically varying VEP topographies that responded to these stimuli were compared to predictions made for them by the Benson-2014 retinotopic atlas (Benson, Butt, Brainard, & Aguirre, 2014).

### Procedure

Participants observed a series of visual stimulation protocols designed to elicit a pattern-pulse mVEP, a pattern-reversal mVEP, and SSVEPs of varying frequencies, the latter of which will be reported separately for a different study. No task was given, other than to maintain steady eye gaze on a central fixation cross, to ensure that we measured VEPs in their most basic form. Participants first observed a 10-minute block of the pulse mVEP, followed by a 20-minute SSVEP block, a 10-minute reversal mVEP block, and finally one further SSVEP block (1 hour total). Opportunities for participants to rest their eyes were provided at regular intervals within each block by requiring a mouse click to continue (continuous stimulus presentations lasted no more than one minute between break opportunities). Participants were, however, required to remain stable on the chin rest throughout each block.

### Stimulus event sequences

**Figure 2.**
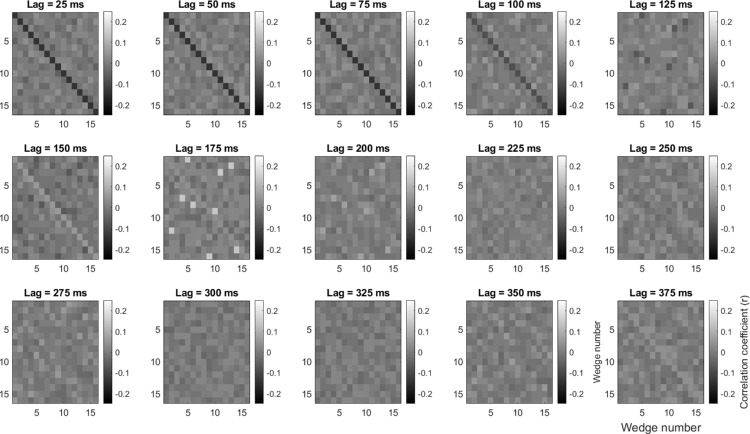
Autocorrelation function across frames (25 ms) of the m-sequence resulting from the omission of closely neighboring pulses, shown both within pulse streams (diagonal) and between pulse streams (off-diagonal). Crucially, the autocorrelation function between pulse streams was flat.

In both mVEP protocols, the 16 wedges followed orthogonal rapid pulse sequences that were governed by 16 time-lagged copies of a zero-autocorrelation series of binary numbers derived from a 2047-frame m-sequence (Baseler, Sutter, Klein, & Carney, 1994; James, 2003). Each frame of this m-sequence corresponded to six monitor refreshes (240 Hz), which yielded frames of approximately 25 ms (40 Hz). For pattern onset, stimulus pulses occurred when “1” appeared in the binary sequence and lasted the full 25 ms. For the remaining “0” frames in the sequence, the background remained gray for 25 ms. To limit the overall pulse speed, the pulse stream was thinned out by first removing consecutive pulses (keeping only the first) and then by removing every second of the remaining pulses, which retained 256 of the original 1024 pulses extending over a time period of 54.6 seconds, similarly to what has been done elsewhere (Mohr et al., 2024; Vanegas, Blangero, & Kelly, 2013). This thinning procedure introduced a small amount of autocorrelation into the pulse streams (see Figure 2) but crucially this was present only within pulse streams and not between pulse streams. The impact of this autocorrelation can be seen most clearly in Figures 9A and 9B where an artifactual component appears at stimulus onset but with a magnitude approximately 12 times smaller than the subsequent stimulus-evoked component. This same pulse train was also used for the pattern-reversal mVEP except that when “1” appeared in the m-sequence, the check pattern reversed at the onset of that 25-ms frame and for the remaining “0” frames the check pattern remained unchanged. Although this constitutes a considerable slowing of the reversal rate compared with what is typical in the literature, this was done to make a direct comparison with the pulse mVEP, the pulse rate of which had been determined previously based on pilot data (Vanegas et al., 2013). We expand on the impact that this reversal-rate choice may have had in the discussion. In both cases, the procedure was repeated 10 times with different m-sequences yielding 2560 pulses/reversals per location. The fixation cross appeared on the screen one second before the first pulse in each repetition, and the participant clicked the mouse button to proceed to each subsequent repetition.

### Data acquisition and processing

EEG data were recorded at 512 Hz by an ActiveTwo Biosemi system with 128 scalp electrodes following the Biosemi ABC layout (Biosemi, Amsterdam, Netherlands) and four external flat-faced electrodes placed above and below the left eye, and on the left and right outer canthi. Eye gaze and blinks were monitored via these four electrooculogram electrodes as well as an Eyelink Plus 1000 Tower system (SR Research, Kanata, ON, Canada) recording at 1000 Hz.

EEG processing was carried out using a combination of inhouse Matlab scripts (MathWorks, Inc., Natick, MA, USA) and EEGLAB routines (Delorme & Makeig, 2004). Continuous data were low-pass filtered by convolution with a 77-tap hanning-windowed sinc function with a 3-dB corner frequency of 35.3 Hz and 50 Hz attenuation (mains) of 83.5 dB. Bad channels (identified by visual inspection of each block) were then interpolated using EEGLAB's *eeg_interp* function. The data were re-referenced to the average of all scalp channels and low frequency trends were removed by linearly detrending each of a sequence of 0.5-second segments within each block to avoid introducing distortions from high pass filters (Acunzo, MacKenzie, & van Rossum, 2012), as we have done previously (Mohr et al., 2024).

Evoked potential waveforms in the epoch range from −100 ms to 400 ms were generated by regressing each block against the stimulus pulse matrix. Specifically, the pulse/reversal sequence for each location was first resampled to align with the EEG sampling rate, and then for each time sample of the epoch, each channel of EEG data was regressed against a time-lagged version of the pulse matrix (the unlagged pulse matrix corresponded to the time point of 0 ms in the waveform). Grand average waveforms for each mVEP and wedge were then produced by averaging across participants.

### Forward modeling

Forward model predictions of sources from distinct visual cortical areas, based on the stimuli used to elicit the empirical mVEPs, were generated identically to those used previously (Mohr et al., 2024). Briefly, reconstruction and volumetric segmentation of the MNI-ICBM152 non-linear average brain was carried out using Freesurfer (http://surfer.nmr.mgh.harvard.edu/; Dale, Fischl, & Sereno, 1999; Fischl, Sereno, & Dale, 1999; Fischl & Dale, 2000; Salat et al., 2004). The cortical surface area corresponding to each wedge was then estimated in this brain (Grabner et al., 2006) by using the *Neuropythy* Python module (Benson & Winawer, 2018) to fit the Benson-2014 retinotopic atlas (Benson et al., 2014) and define regions of interest for the segments of the visual field encapsulated by each of the 16 wedges for each visual area. This was used to generate a list of voxel coordinates corresponding to the gray matter surface area for each wedge in each visual area.

The Matlab toolbox Fieldtrip (Oostenveld, Fries, Maris, & Schoffelen, 2010) was then used to generate a conductivity head-model based on this brain (boundary element model). This was used to derive scalp topographies resulting from dipoles located at each of the voxels determined by the above regions of interest, with orientations constrained by the surface normal of the corresponding cortical surface area. These single-voxel topographies were then averaged over all voxels corresponding to each wedge (assuming uniform activation across all voxels) to yield the final predicted topographies for each wedge. This whole process was repeated for eight visual areas (V1, V2, V3, V4, TO1, TO2, LO1, and LO2, chosen simply based on availability in the atlas) to yield predictions of their retinotopically mapped topographies for each wedge.

## Results

### Amplitude and SNR

**Figure 3.**
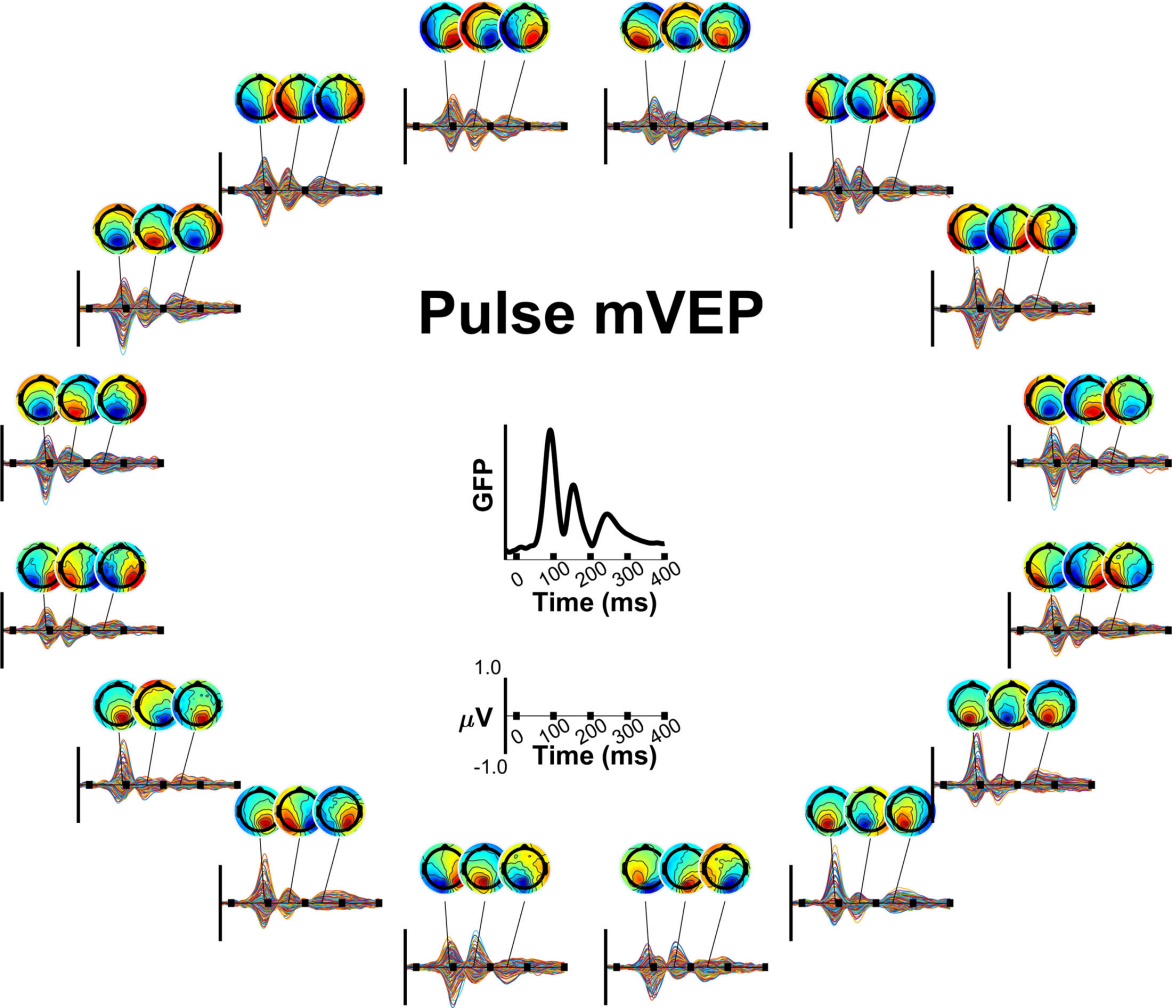
Pulse mVEPs. Butterfly plots (one trace per channel) and topographies are shown by stimulus location around the circle and global field power (GFP) is shown averaged across locations in the center. The axis below the GFP plot shows the axis limits for all plots in the surrounding circle.

**Figure 4.**
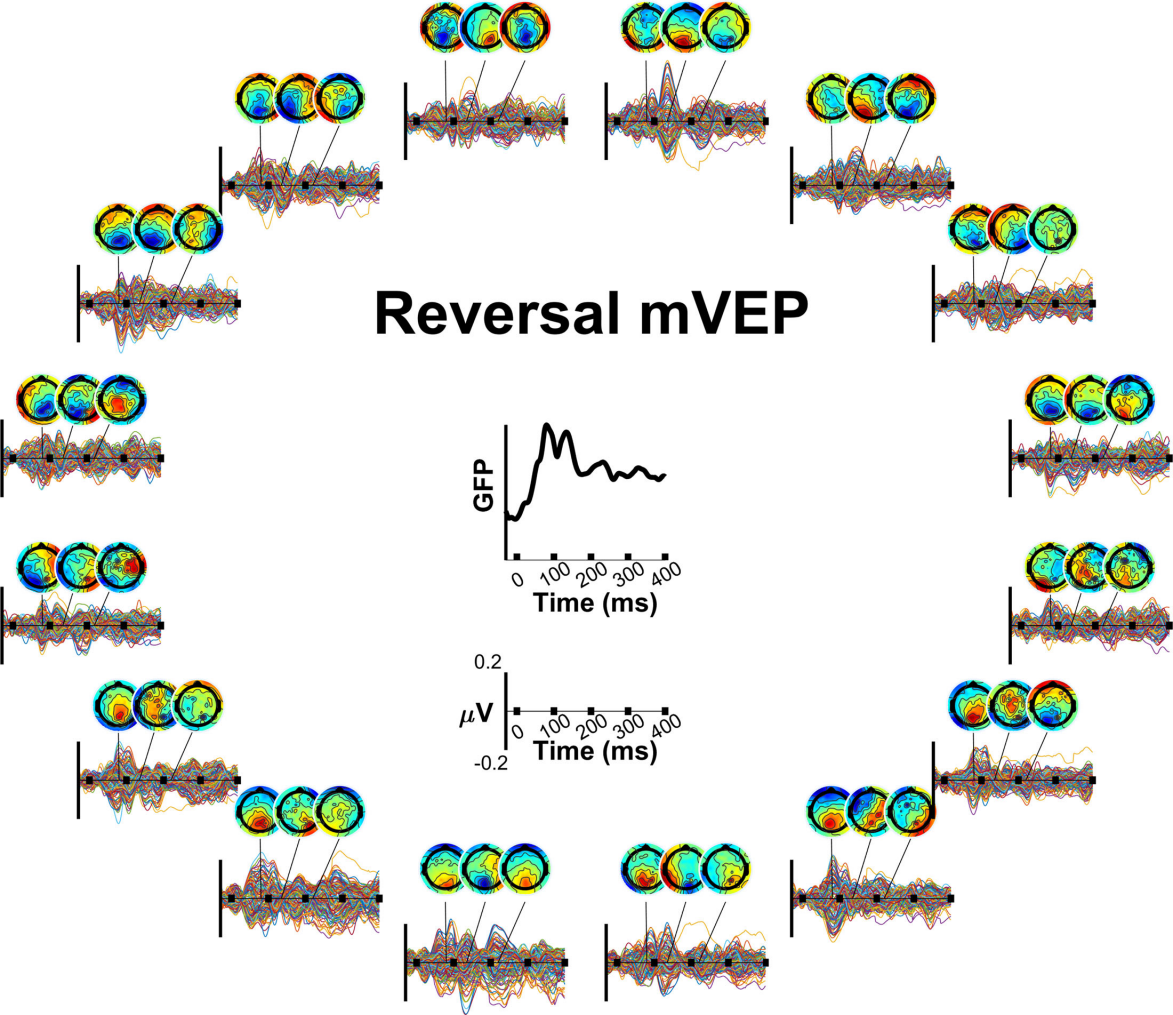
Reversal mVEPs. Butterfly plots (one trace per channel) and topographies are shown by stimulus location around the circle and GFP is shown averaged across locations in the center. The axis below the GFP plot shows the axis limits for all plots in the surrounding circle.

Figures 3 and 4 show the grand average waveforms for the pulse and reversal mVEP, respectively. Comparing the grand average global field power (GFP) of the two signals between 80 ms and 90 ms, stimulus locked, it was 3.2 times larger in the pulse mVEP, whereas GFP between −50 ms and 0 ms was comparable (21% higher in the pulse mVEP). As such, SNR was higher in the pulse mVEP than the reversal mVEP to a similar extent (2.6 times).

### Comparing the pulse and reversal mVEP waveforms

**Figure 5.**
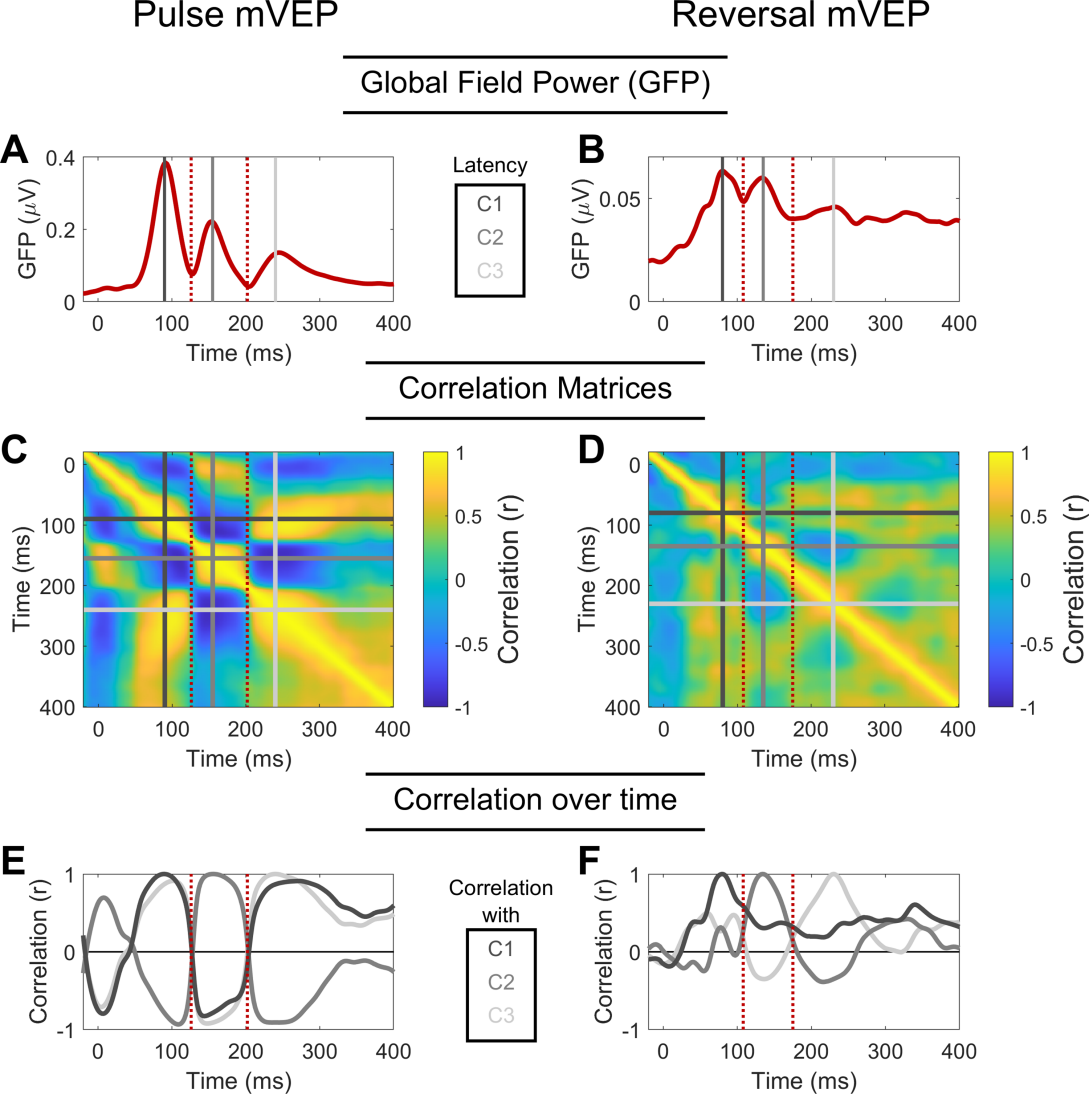
Global field power for (**A**) the pulse mVEP and (**B**) the reversal mVEP. The three gray lines highlight the latency of the three component peaks (C1, C2 and C3). The red dotted lines highlight the dips in GFP between the C1 and C2, and between the C2 and C3. (**C**, **D**) Correlation matrices quantifying the similarity in retinotopically-varying topographic profiles between any pair of time points in (**C**) the pulse mVEP and (**D**) the reversal mVEP. The gray lines correspond to the latency of the C1, C2 and C3 components identified in the GFP plots and the dotted red line to the dip in GFP between C1 and C2. (**E**, **F**) Line plots of the rows of the correlation matrices outlined by the differently shaded gray lines, which correspond to the three components of the (**E**) pulse mVEP and (**F**) reversal mVEP. The dotted red line highlights the dip in GFP between the C1 and C2.

**Figure 6.**
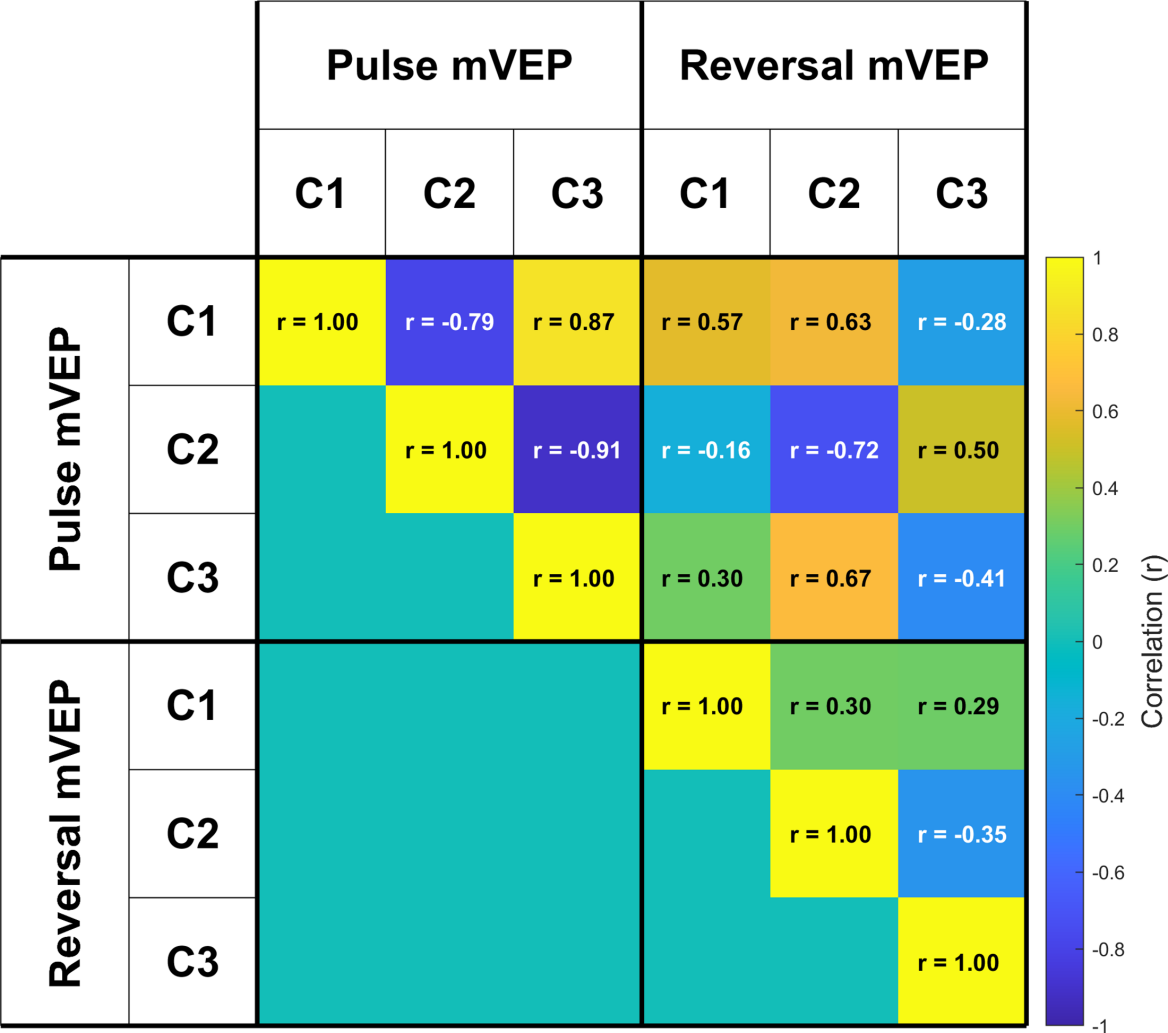
Correlations between mVEP components in terms of retinotopic patterns of topography across stimulus location.

A series of three components could be identified in each signal (Figures 3 and 4), peaking at approximately 90 ms, 155 ms and 240 ms in the case of the pulse mVEP and 80 ms, 135 ms and 230 ms in the case of the reversal mVEP. The first of these components in the pulse mVEP is classically referred to as the “C1” while the first component of the reversal mVEP is usually referred to as the “N75”, both of which are usually observed to reverse in polarity for stimuli presented in the upper versus lower visual fields, as observed here. For convenience, we will refer to the first component of both the pulse and reversal mVEP as C1, and the subsequent components as C2 and C3. A cross-correlation across time is shown for each mVEP in Figure 5. In this analysis, a vector of grand average VEP amplitudes across all electrodes and stimulus locations was formed at each time point, and these vectors were correlated for each pair of time points. The subset of these correlations that correspond to pairs of time points when the component peaks were observed are shown in Figure 6 (both within and between the pulse and reversal mVEPs).

**Figure 7.**
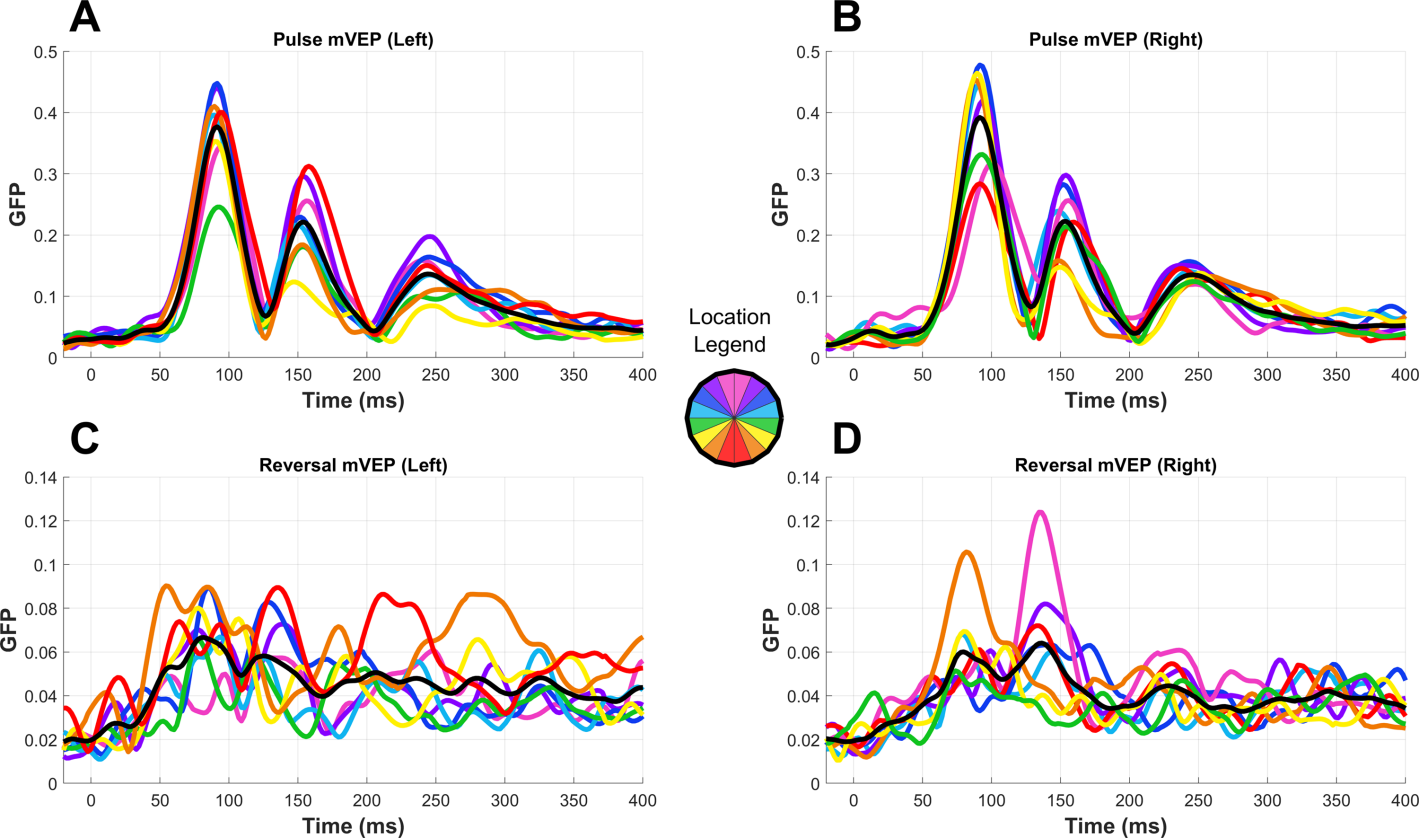
GFP by location for the pulse (**A**–**B**) and reversal (**C**–**D**) mVEPs. For clarity, separate panels are shown for stimuli in the left (**A** and **C**) and right (**B** and **D**) hemifield. Note that the y-axis scales are different, reflecting the different magnitudes of the pulse and reversal mVEP.

Figures 5 and 6 show a strong correlation among all three components for the pulse mVEP, with C2 negatively correlating with both C1 (*r* = −0.79) and C3 (*r* = −0.91), and C1 and C3 correlating positively (*r* = 0.87). In the reversal mVEP, by contrast, while C2 and C3 were negatively correlated with each other (*r* = −0.35) as in the pulse mVEP, the C1 showed a different trend, being positively correlated with both C2 (*r* = 0.3) and C3 (*r* = 0.29) where neither correlation constituted a local extremum in the cross-correlation profile as was the case for the pulse mVEP (Figures 5E and 5F). Thus, although the pulse mVEP consisted of three consecutive components that were near-identical in their retinotopically-dependent scalp topographies aside from polarity flips, the reversal mVEP showed a C1 component that was not strongly correlated with the subsequent components. Comparing between the pulse and reversal mVEPs (top right quadrant of Figure 6), while the pulse and reversal C1s were positively correlated (*r* = 0.57), the pulse and reversal C2s were negatively correlated (*r* = −0.63), as were the C3s (*r* = −0.28). Instead, the reversal C2 and C3 were positively correlated with the pulse C1 and C2 (*r* = 0.63 and *r* = 0.5, respectively). In other words, the response pattern typified by the pulse mVEP C1 and C2 was exhibited in the reversal mVEP later, in the C2 and C3. Accordingly, we also observed differences between the pulse and reversal mVEP in how the waveforms transitioned from C1 to C2. In the pulse mVEP, the low point in GFP between C1 and C2 coincided with a low point in correlation of topographic profiles with C1 (Figure 5C, 5E) that was not significantly different from zero (*r* = 0.11, *p* > .1). By contrast, in the reversal mVEP, the low point in GFP between C1 and C2 was associated with a significant positive correlation of activity with C1 (*r* = 0.44, *p* < 0.01), which was significantly higher than for the pulse mVEP (*F*(1,12) = 4.79, *p* < .05; Figures 5D and 5F). A different pattern of results was observed during the transition between C2 and C3. In this case, the correlations during the low-point in GFP were not significantly different between the pulse and reversal mVEP (*F*(1,12) = 2.23, *p* > 0.1) or significantly greater than zero (*F*(1,12) = 3.53, *p* > 0.05). Thus, although the pulse mVEP exhibited “silent” moments of low GFP between each component peak that were associated with zero-correlation with the preceding component peak, this “silent moment” was present in the reversal mVEP only between the C2 and C3. Observing GFP profiles in each stimulus location separately, the pulse mVEP exhibited three peaks that were clearly aligned at every location whereas there was no clear alignment in GFP peaks across locations for the reversal mVEP (Figure 7). Taken together, these patterns are suggestive of a unitary triphasic response in the pulse mVEP (i.e., a response from the same source(s) that consists of three consecutive phases), and, in contrast, a more complex superposition of responses in the reversal mVEP.

### Visual area modeling

**Figure 8.**
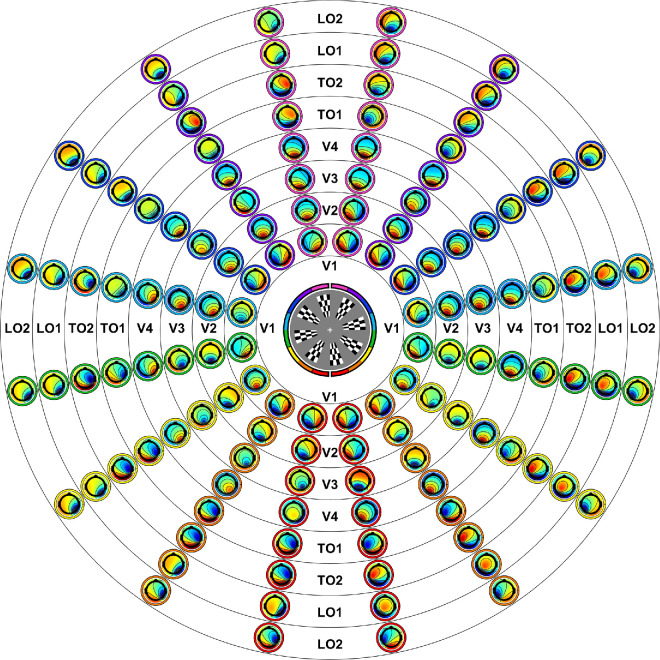
Predicted topographies for each visual area and stimulus location (all assuming a surface negative potential at the cortical surface for consistency).

**Figure 9.**
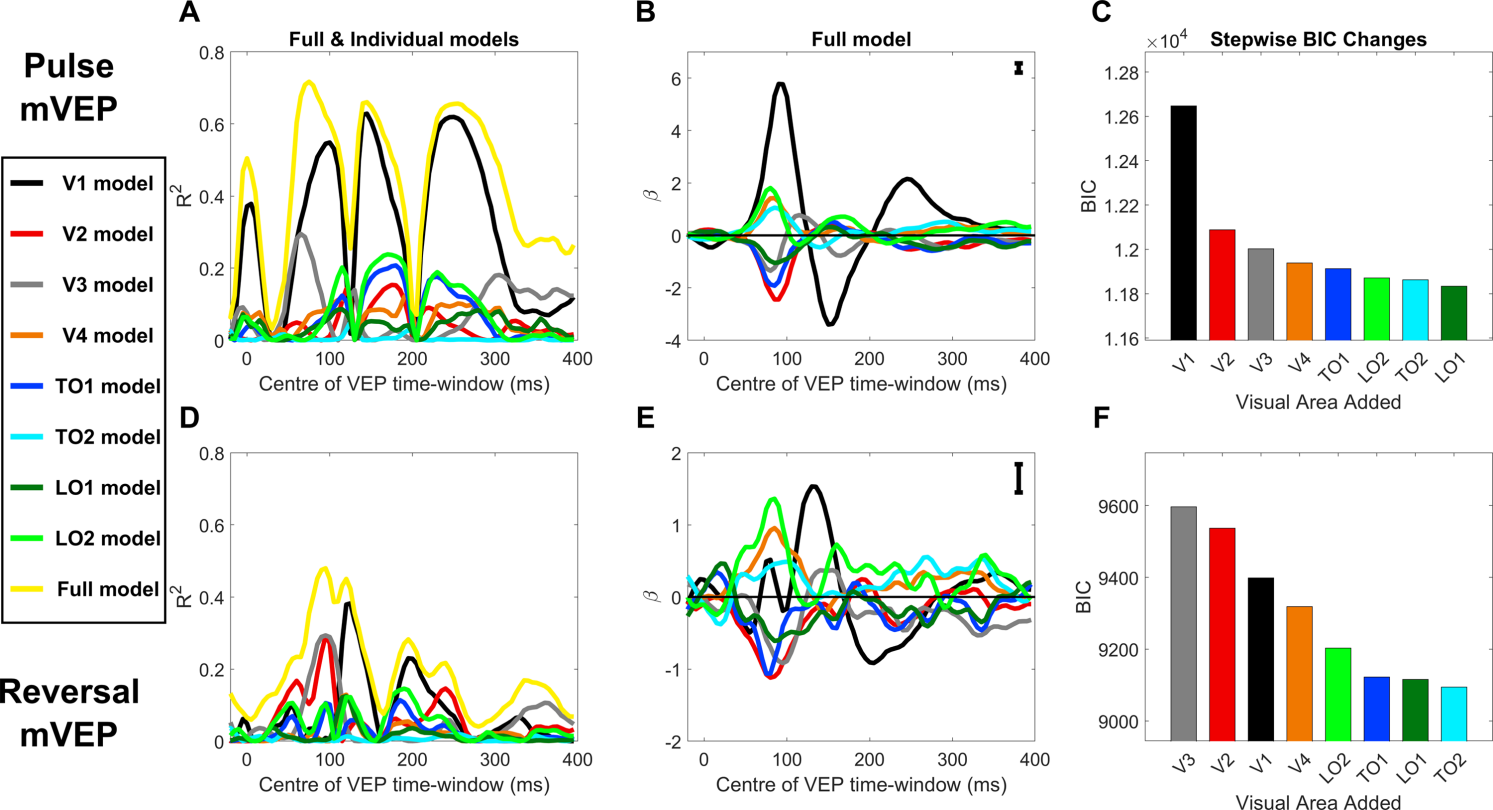
Visual area models of the pulse (**A**–**C**) and reversal (**D**–**F**) mVEPs. (**A**, **D**) Explained variance from each visual area is shown as a function of time in each individual area model, as well as in the full model including all eight visual areas. (**B**, **E**) The time course of beta-weights in the eight-area model. Representative error bars are shown in the upper right corner of these plots. These are based on the average standard error at each time point and for each visual area, calculated from a bootstrap sample of participants (*N* = 1000). Note the different y-axis scales that are due to the different magnitudes of the pulse and reversal mVEPs. (**C**, **F**) Sequence of BIC values encountered in a stepwise regression procedure whereby visual areas were added to the model one at a time, choosing the visual area at each step that best improved BIC.

To better understand the visual sources underpinning these differing trends between the pulse and reversal mVEP, we applied a retinotopically constrained source analysis to the two mVEPs. Using the retinotopically varying forward models derived from the Benson-2014 atlas, the relative contributions to the pulse and reversal mVEP were estimated for V1, V2, V3, V4, TO1, TO2, LO1 and LO2. Each visual area predicted a topography for each stimulus location (Figure 8). Thus a regressor was formed for each visual area with 2048 elements (128 electrodes by 16 locations). These regressors were used to predict grand average mVEP topographies across stimulus locations for each of a sequence of averaged time windows of width 10 ms sliding in 5-ms steps to span a stimulus-locked epoch of −100 ms to 400 ms. All regressors and the two mVEPs were converted to *z*-scores before carrying out the regression (means and standard deviations for the mVEPs were derived from the full epoch). Models were run for each visual area individually, and a “full model” was constructed by a stepwise regression approach with forward selection based on the VEP from 80 to 90 ms. This included all eight visual areas as regressors in the full model for both the pulse and reversal mVEPs (Figures 9C and 9F).

The results of these regressions are shown in Figure 9. For both the pulse and reversal mVEP, the greatest proportion of variance explained by the full (eight-area) model was achieved during the C1 time frame where 72% was explained for the pulse mVEP between 70 and 80 ms (Figure 9A), and 48% was explained for the reversal mVEP between 90 and 100 ms (Figure 9D). In the case of the pulse mVEP, most of this could be accounted for individually by V1, which explained 43% of variance between 70 and 80 ms in its single-area model (climbing to 54% between 90 and 100 ms). The next most predictive area was V3, which on its own accounted for 29% of variance between 65 and 75 ms. V1 remained the best predictor for each of the subsequent waves of the pulse mVEP (Figure 9A). By contrast, very little variance in the C1 time frame of the reversal mVEP was accounted for by V1, which explained 3.5% of variance at its best between 75 and 85 ms. Eight-fold more variance was accounted for by either V2 or V3 individually (29% each) between 90 and 100 ms. However, after this initial dominance of extrastriate areas in the reversal mVEP, V1 became the best predictor by 120 ms, individually explaining 38% of variance between 120 and 130 ms, which was only incrementally improved in the full model including all eight visual areas, which predicted 42% of variance in the same time range.

These patterns, whereby V1 was dominant throughout the pulse mVEP but did not become dominant in the reversal mVEP until after an initial wave of extrastriate dominance, can be seen in the time course of beta weights for the full 8-area model (Figures 9B and 9E). These show that V1 underwent a triphasic response in the pulse mVEP with much smaller contributions from areas beyond V1 (Figure 9B). By contrast, in the reversal mVEP, there was an initial activation across all visual areas that was smallest in V1, and this was followed by a biphasic response in V1 of similar form to that of the pulse mVEP but delayed by approximately 50 ms. Notably, during the initial component (C1), there was an approximately even split of positive and negative polarity amongst the 8 visual areas, and this variation in sign was matched between the pulse and reversal mVEP despite their differing topographies (Figures 9B and 9E). In other words, despite different retinotopic patterns of topographies being present in the C1 component of the pulse and reversal mVEP, a similar profile of extrastriate responses was attributed to them both, with the difference lying in the relative magnitude of the contribution from V1. Moreover, this consistency between the two mVEPs, along with the relatively small standard errors of beta weights (Figures 9B and 9E), calculated from bootstrap samples of participants (*N* = 1000), reflect good internal reliability of these beta traces.

### Component correlations across locations and visual areas

**Figure 10.**
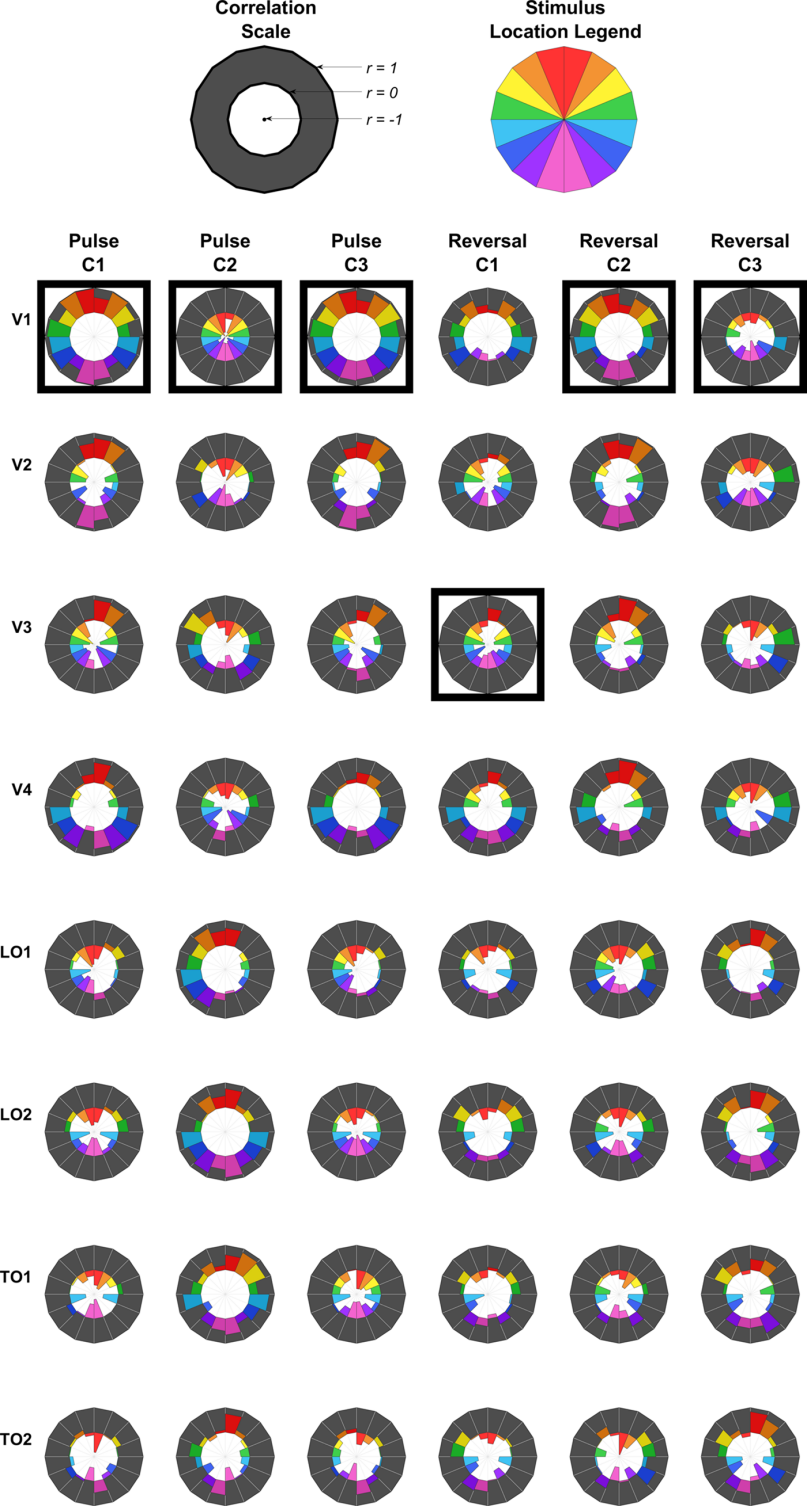
Correlations between mVEP component topographies and each visual area prediction as a function of stimulus location. V1 Correlations are consistent in their direction across locations for all components of the pulse mVEP, but this is not so for the reversal C1. For each component, the visual area that has the largest average correlation across locations is highlighted with a black square.

The above analyses identified correlations among the C1, C2 and C3 within each of the pulse and reversal mVEPs by inspecting retinotopic patterns in their topographies, and also identified the likely cortical sources of these components by identifying which visual areas provide the best overall match of their retinotopic patterns of topography. One notable observation was that although the pulse and reversal C1s differed greatly in the size of the contribution from V1, a similar pattern of contributions from extrastriate areas was present in both (Figures 9B and 9E). Therefore we next sought to compare the C1, C2, and C3, both within and across the pulse and reversal mVEPs, in a way that reflects these patterns of contributions across visual areas by comparing the retinotopic patterns of correlations between components and visual area predictions. Specifically, we calculated the correlation between all six components (C1, C2, and C3 for the pulse and reversal mVEPs) and each visual area for all 16 stimulus locations separately. These correlations are shown in Figure 10. This showed that while V1 was uniformly positively correlated with the pulse C1 at all locations (and negatively correlated with the pulse C2), these correlations were not uniformly positive for the reversal C1, but mostly were for the reversal C2 (Figure 10). This indicates that similar V1-dominated cascades of visual area responses were present in the pulse and reversal mVEP, but although it began at the C1 for the pulse mVEP, it began at the C2 for the reversal mVEP.

## Discussion

In this study, we investigated the cascade of visual sources underlying the multifocal visual evoked potential (mVEP) for pattern pulses and pattern reversals by comparing retinotopically varying topographies with predictions from the Benson-2014 retinotopy atlas for a suite of visual areas. This replicated our previous finding that the pulse mVEP is dominated by a source in V1 throughout its time course (Mohr et al., 2024). The pulse mVEP consisted of a sequence of three highly similar components, attributed here to a dominant triphasic response in V1 since V1 accounted for the majority of its variance and the estimated V1 waveform had a magnitude more than twice that of any other visual area and a triphasic morphology. The strong correlation among these three components, together with their remarkable temporal alignment across stimulus locations and moments of minimal activity as one component transitions to the next, make a strong case that the pulse mVEP is dominated by this triphasic V1 response as any strong overlapping activity would likely not share the temporal alignment of these peaks and would therefore obscure them, such as in the transient VEP where the C1, P1 and N1 overlap and obscure one another (Luck, Woodman, & Vogel, 2000). A different cascade of visual areas underpinned the reversal mVEP. The first component received a smaller contribution from V1 than from any extrastriate area and this component did not have the same correlational structure with later components as in the pulse mVEP. Instead, V1 became dominant from the second component onwards, beginning a biphasic response at that point. Thus, although the pulse mVEP was characterized by a dominant triphasic response in V1, the reversal mVEP was characterized by a delayed biphasic response in V1 that followed an initial activation of extrastriate areas. Notably, despite this large difference in the contribution of V1 to the C1 components of the pulse and reversal mVEP, there was a similar profile of extrastriate contributions in the visual area models, which likely contributes to the topographies remaining quite well correlated (*r* = 0.6). Thus it appears that the pulse and reversal mVEP may contain similar patterns of initial extrastriate activation but differ markedly in the involvement of V1, which massively dominates over this extrastriate activity in the pulse mVEP during that initial response phase.

As mentioned in the Introduction, a range of differences in response properties have previously been demonstrated between the mVEPs, including greater contrast saturation in the reversal mVEP (Maddess et al., 2005), a greater center-versus-periphery SNR advantage in the pulse mVEP (Hoffmann et al., 2003; Klistorner & Graham, 2005; Souza et al., 2012), and a greater SNR advantage from temporal and spatial stimulus sparsity in the pulse mVEP (Fortune et al., 2009; James et al., 2005; Maddess et al., 2005). In light of our current findings, it is interesting to consider whether and how these differences in response properties may arise from the differences in processing cascade across visual areas we observed here. Notably, some of the above differences are echoed in comparisons between V1 and extrastriate areas in a way that is consistent with our finding of V1 dominance in the pulse mVEP. For example, extrastriate areas have more saturated CRFs than V1 (Tootell et al., 1998), mirroring the more saturated CRFs in the reversal mVEP compared with the pulse mVEP. Moreover, the surround suppression field is broader in extrastriate areas than in V1 (Spillmann, Dresp-Langley, & Tseng, 2015), which is consistent with the greater SNR advantage from higher spatial stimulus sparsity in the pulse mVEP compared with the reversal mVEP because a smaller suppression field in V1 would make the V1-dominated pulse mVEP better suited to benefit from increased spatial sparsity. Thus our finding of a more dominant V1 contribution in the pulse mVEP than the reversal mVEP is consistent with differences in their response properties.

Another possible difference in the pulse and reversal mVEPs that could account for the above differences in response properties is the relative balance of activity generated in the magnocellular and parvocellular pathways (Klistorner & Graham, 2005). Compared with parvocellular neurons, magnocellular neurons tend to prefer higher temporal frequencies, lower spatial frequencies, have more saturated CRFs, and are relatively more abundant for peripheral eccentricities (Derrington & Lennie, 1984; Merigan & Maunsell, 1990; Shapley & Perry, 1986). An account whereby the reversal mVEP is relatively more subserved by the magnocellular pathway (during its first wave) would align with these observations. Although the parvocellular and magnocellular pathways converge in V1 (Callaway, 2005; Nealey & Maunsell, 1994), seemingly at odds with the much smaller V1 contribution to the C1 component of the reversal mVEP than the pulse mVEP, magnocellular activation typically precedes parvocellular activation by about 20 ms (Schmolesky et al., 1998) and this “magnocellular advantage” is thought to allow it to carry an initial crude representation of the visual scene that can be fed back to V1 to inform afferent parvocellular responses (Bullier, 2001; Laycock, Crewther, S. G., & Crewther, 2007). The generation of this crude representation by the magnocellular pathway also may not feature as prominently in EEG recordings as parvocellular activity due to the much lower prevalence of magnocellular afferents from the lateral geniculate nucleus, which are outnumbered by parvocellular afferents by approximately 8:1 (Perry, Oehler, & Cowey, 1984). Indeed, response properties of the C1 and related VESPA component are suggestive of generation predominantly by parvocellular neurons (Foxe et al., 2008; Lalor & Foxe, 2009). If the C1 component does indeed reflect activation of V1 by parvocellular afferents then it could incorporate feedback from a crude representation generated rapidly by the magnocellular pathway (Bullier, 2001). In the case of pattern reversal, it may be possible for feedback from this crude representation to more accurately guide afferent parvocellular activity because the overall form of the stimulus does not change. In this way, pre-existing perceptual templates may allow for more accurate feedback, potentially reducing some of the usual processing requirements for perception (Fahrenfort, Scholte, & Lamme, 2008). In principle, this could render the typical large V1 response usually observed in the C1 component less necessary, which could explain why the C1 component of the reversal mVEP was not dominated by V1 as in the pulse mVEP. Exploiting this environmental regularity to facilitate a more efficient processing pipeline is in keeping with efficient coding theory, which purports that the visual system takes account of statistical regularities of the environment to process the visual scene in efficient ways that avoid redundancies (Simoncelli & Olshausen, 2001). Taken together, these considerations raise a hypothesis that the pulse and reversal mVEPs reflect processing along different visual pathways initially, but later converge in V1 because the C2s of both mVEPs were best explained by V1 activity. Although this interpretation is highly speculative, we raise it merely to offer a possible rationale for why the processing pipeline of the pulse and reversal mVEP might differ, which in any event is strongly implied by their different cortical source sequences demonstrated here, and their different dependencies on stimulus contrast, eccentricity, and sparsity discussed above.

Ultimately, the mechanistic underpinnings of these differences between the pulse and reversal mVEP in the cascade of visual area activations remain to be characterized fully. Nevertheless, the present data point to a crucial difference being the time at which V1 activity becomes dominant. Such differences do not impact the clinical use of these signals to identify conditions affecting the optic nerve such as optic neuritis and glaucoma (Graham et al., 2005; Hood et al., 2000) because these conditions would have upstream effects in the visual cortex regardless of the neural mechanisms involved. In fact, if these differences in cortical sources result in different profiles of SNR across the visual field, then the two protocols can be used together so that weak signals in one mVEP can be compensated by the other (Klistorner & Graham, 2005; Souza et al., 2012). However, differences between the pulse and reversal mVEP should be taken into account when using them as clinical biomarkers of visual function in conditions affecting the central nervous system (Laycock et al., 2007) such as in schizophrenia (Yamada, Yukawa, Taketani, Matsuura, & Hara, 2011), autism (Jackson et al., 2013; Sutherland & Crewther, 2010) and multiple sclerosis (Pihl-Jensen, Schmidt, & Frederiksen, 2017).

One important distinction between our implementation of the reversal mVEP and its typical implementation in the literature is the reversal rate. Usually, the reversal mVEP is implemented with a one-to-one mapping between m-sequence step and monitor refreshes, which with typical refresh rates of 60 Hz or 75 Hz incurs reversal rates of approximately 35 Hz per location on average. Here, to make the reversal rate the same as the pulse rate of the pulse mVEP, we thinned out the m-sequence such that the reversal rate was approximately 5 Hz per location on average. This is likely the reason why the SNR of the reversal mVEP here was approximately three times lower than the pulse mVEP. Indeed, comparisons between reversal mVEP at the typical fast rate and pulse mVEP at the typical slow rate often find similar SNRs (Fortune et al., 2009; Souza et al., 2012), and even studies reporting an SNR advantage for the pulse mVEP find that advantage to be smaller in magnitude to what we observed here (James, 2003; Klistorner & Graham, 2005). Beyond this SNR consideration, the slower-than-usual reversal rate means that our finding that V1 does not dominate the first component of the reversal mVEP may not extend to the faster reversal rate that is typically used. Indeed, Fortune and Hood (2003) compared fast and slow multifocal reversal VEPs along with transient reversal VEPs (5 Hz overall event rate) and found that the slow multifocal and transient reversal VEPs were similar to one another—both showing, for example, a large amplitude discrepancy between the upper and lower field that did not show polarity reversal—and different to the fast reversal mVEP, which showed similar amplitudes and polarity reversal between the upper and lower field. However, it should be noted that their finding of a lack of polarity reversal between the upper and lower visual field in the slow reversal conditions conflicts with other reports of transient reversal VEPs (Di Russo et al., 2005), with one methodological difference being their use of full hemifield stimuli in eliciting the transient reversal VEP (Fortune & Hood, 2003). Notwithstanding possible differences in the reversal VEP across different reversal rates, our finding that extrastriate sources underpin the first component of the reversal mVEP nevertheless challenges the usual assumption that reversal VEPs have a dominant source in V1 because this assumption is underpinned by the same set of rationales for the transient and multifocal reversal VEPs: source modeling and polarity reversal between the upper and lower visual field (Barnikol et al., 2006; Di Russo et al., 2005; Hood et al., 2006; Slotnick et al., 1999; Zhang & Hood, 2004). Although it is possible that the reversal mVEP implemented here adopts an intermediate reversal rate between the typical fast reversal mVEP and the slow transient reversal VEP and that this explains the discrepancy in sources, this would need to be demonstrated in future research applying the same methods as used here.

The primary dynamic underlying the analyses in this paper was retinotopic changes in VEP topography. Indeed, this has classically been a central driver for inferring V1 sources for signals by virtue of their polarity reversing between the upper and lower visual field. However, this simple heuristic is not sufficient to exclusively diagnose a V1 source (J. M. Ales et al., 2010; Mohr et al., 2024). V1, V2, V3 and both the pulse and reversal mVEP showed this signal pattern and topographies in the C1 component of the pulse and reversal mVEP appeared rather similar on visual inspection, despite being ascribed very different levels of V1 contribution in the model. This reflected subtle differences in topography at select stimulus locations both between the pulse and reversal mVEP, and among visual areas. One example of this was that although both the pulse and reversal mVEP inverted in polarity between the upper and lower visual field, the topography was slightly more ipsilateral in the upper visual field than the lower visual field for the pulse mVEP but not so for the reversal mVEP. This was also true of V1 predictions and not of V2 and V3 predictions. Another example was that pulse mVEP topography became more lateralized as stimuli were presented closer to the vertical meridian whereas lateralization of the reversal mVEP did not change much. Again, this lateralization on approach to the vertical meridian is also predicted by V1 whereas V3 predicts little such lateralization. Features such as these are what underpin the robustness of retinotopically constrained source estimation (RCSE) because they allow cortical sources that might provide similar topography predictions at one retinotopic location to be distinguished based on their divergent predictions at another location (J. Ales et al., 2010; Hagler, 2014; Hagler et al., 2009; Hagler & Dale, 2013). However, there are important limitations to this approach. First, it relies on visual sources predicting patterns of topographies that change dramatically across the visual field in an idiosyncratic manner from one visual area to the next. Although this is the case for the visual areas investigated here, each of which predicted a unique pattern of topography changes across retinotopic space, it may not be true of every visual area. Another limitation of the approach used here is that it relies on a grand average anatomy (MNI) and retinotopy atlas (Benson et al., 2014) rather than measuring both on an individual basis, which would more accurately predict retinotopic patterns of topography change on an individual basis, though see Poncet and Ales (2023) for competitive source estimation performance achieved with an average-based EEG atlas compared with individual MRI-informed source estimation when retinotopic constraints were not in use. Despite the successful application of average-based approaches, it is clear that the use of grand averages smooths out some of the idiosyncratic folding patterns that distinguish visual areas and also leads to slight discrepancies with recorded grand average EEG topographies that retain the footprints of those idiosyncratic folding patterns, though notably, the approach of Poncet and Ales (2023) does not suffer the latter problem. This increases the requirement for visual areas to predict dramatic topographic changes that remain distinguishable following these transformations. Other RCSE approaches use individual fMRI (J. Ales et al., 2010; Hagler, 2014; Hagler et al., 2009; Hagler & Dale, 2013), which avoids this problem but involves an additional layer of uncertainty due to noise in fMRI measurements and increases the practical burden of implementing RCSE by requiring fMRI recordings for each participant. Either way, by exploiting different retinotopic profiles of geometry between different visual areas, RCSE can harness strong constraints for source estimation that help to distinguish closely neighboring visual areas and help to advance our understanding of the cortical sources underpinning visual evoked potentials.

## Conclusions

In this study, we used grand average retinotopy from functional magnetic resonance imaging to study the cortical sources underpinning the pulse and reversal mVEP. In doing so, we found that although the pulse mVEP was dominated by activity in V1 throughout its time course, the reversal mVEP was initially dominated by activity in extrastriate areas with V1-dominated activity following afterwards. This suggests a substantive difference in the visual processing cascade for stimuli that are pulsed on and off compared with stimuli that are reversed in contrast pattern but remain otherwise unchanged. Although these differences do not impact the clinical use of mVEPs to identify visual field defects where the pathophysiology lies before entry to the cortex, studies that use the mVEP to study cortical visual (dys-) function should take these differences into account.
